# The Inner Road to Happiness: A Narrative Review Exploring the Interoceptive Benefits of Exercise for Well-Being

**DOI:** 10.3390/healthcare13161960

**Published:** 2025-08-10

**Authors:** Laura Barca

**Affiliations:** Institute of Cognitive Sciences and Technologies, National Research Council, 00185 Rome, Italy; laura.barca@istc.cnr.it; Tel.: +39-6-44595312

**Keywords:** health promotion, interoception, physical exercise, mental health, well-being

## Abstract

Background: Interoception, the multifaceted perception of internal bodily signals, is crucial for homeostasis, emotional regulation, and overall well-being. Physical exercise significantly influences interoceptive mechanisms through its varied physiological, neurobiological, and psychological impacts. Despite its potential to enhance this internal sensing across its dimensions and foster adaptive behaviors like self-regulation, exercise remains an underutilized therapeutic approach. Objective: This narrative review explores the current understanding of the interplay between exercise and interoception, examining its resulting impact on both mental and physical health. Method: A comprehensive literature search was conducted on PubMed using keywords such as “interoception,” “exercise,” and “well-being.” Article selection prioritized empirical studies, reviews, and influential theoretical papers. The synthesis of the literature was performed through a thematic analysis, structured around three primary mechanisms: physiological changes, neurobiological adaptations, and psychological benefits. Key Findings: Engaging in exercise improves interoceptive function by inducing physiological changes, fostering neurobiological adaptations, and yielding psychological advantages such as reduced stress. This enhancement in internal bodily sensing, encompassing its various dimensions, and promotion of adaptive behaviors has notable consequences for well-being. Conclusions and Future Directions: Exercise presents a valuable and readily available means to enhance interoceptive processing and encourage adaptive behaviors, with substantial positive implications for well-being throughout life. Future studies should focus on identifying the most effective exercise approaches tailored to individual requirements and exploring their specific impact on different interoceptive dimensions. Integrating exercise into clinical treatment plans and public health strategies offers a promising path to substantially boost well-being.

## 1. Introduction

Human well-being is a multifaceted construct, profoundly influenced by both external circumstances and internal states. In today’s fast-paced and increasingly complex world, marked by rising rates of chronic stress, anxiety, and mental health challenges (e.g., as widely observed during global crises like the COVID-19 pandemic [1,2,3]), the search for sustainable well-being strategies has become paramount. There is a growing recognition that solely addressing external factors is insufficient; true resilience and health are rooted in our capacity for self-awareness and the perception of our internal physiological landscape. This internal journey of self-discovery and regulation, which we refer to as the “inner road,” is the focus of this article’s exploration into how it can be positively impacted.

At the core of this concept lies *interoception*, defined as the perception of internal bodily signals, such as heart rate, respiration, and gut sensations. Beyond merely physiological monitoring, interoceptive processes are increasingly recognized as fundamental for crucial processes like homeostasis [4], emotional regulation, and the broader experience of well-being [5]. Contemporary models, such as those proposed by Garfinkel and colleagues [6], offer a nuanced understanding of interoception by outlining various dimensions (e.g., interoceptive accuracy, awareness, or sensibility). *Interoceptive accuracy* refers to the precision with which an individual detects and discriminates internal physiological signals (e.g., heartbeats). This dimension is often objectively measured using tasks that involve counting one’s own heartbeats or perceiving one’s breathing patterns. *Interoceptive awareness* pertains to the metacognitive ability to recognize, interpret, and reflect upon one’s own internal bodily sensations. It involves the subjective perception and conscious knowledge of these signals. *Interoceptive sensibility* denotes an individual’s general tendency or propensity to pay attention to and notice their internal bodily sensations, regardless of their objective accuracy. It is often assessed via self-report questionnaires (see [7]). Maintaining a clear distinction among these dimensions is crucial for fully understanding how different facets of interoception contribute to well-being and how they can be influenced by interventions such as physical exercise. Where appropriate throughout this review, we will specify the particular dimension of interoception being referred to.

Departing from the traditional view of interoception as mere sensory input, the framework of Active Inference [8,9] highlights its dynamic role in shaping our interactions with the world. By integrating interoceptive signals with exteroceptive (vision, touch, hearing, taste, and olfaction) and proprioceptive information within a hierarchical brain architecture, we construct a rich and nuanced representation of our internal and external states [10]. This process is underpinned by predictive coding [11], where the brain constantly generates and updates predictions about its internal and external states. Consequently, interoceptive signals play a pivotal role in shaping our perceptions, emotions, and decision-making processes. In essence, our internal bodily states are not merely passive recipients of sensory information, but active participants in shaping our conscious experience. This perspective is increasingly utilized to model subjective well-being and resilience, suggesting that maintaining well-being involves minimizing prediction errors about one’s internal state and actively engaging with the world to reduce interoceptive uncertainty, thus fostering stable and desirable internal physiological states [12,13,14].

This active interplay between the various dimensions of interoception and other sensory modalities has profound implications for our overall well-being. A deeper understanding of these interoceptive processes reveals that they play a key role in shaping not only our immediate perceptions but also our broader mental and emotional states. For example, the ability to accurately perceive and be aware of our internal bodily states can significantly influence mental health outcomes, decision-making, and stress management. However, this process can be disrupted. Individuals with anxiety disorders, for example, often experience heightened interoceptive sensibility to bodily sensations that may be perceived as threatening [15]. Dysregulations can also arise from noisy or poorly differentiated interoceptive signals, which makes it difficult to distinguish bodily and affective states. This altered interoception might be implicated in the development of maladaptive coping strategies for managing uncertain bodily states, such as restrictive eating [16] or self-injury [17]. These self-directed actions, while seemingly maladaptive, can be viewed as attempts to achieve a more coherent sense of self within the context of an uncertain internal milieu. This perspective underscores the pivotal role of interoception not only in shaping our experience of the world but also in driving our behaviors, both adaptive and maladaptive.

Despite extensive research on the link between altered interoceptive processes and maladaptive behaviors, the idea of fostering these processes to promote positive well-being has received comparatively less attention. Exploring this aspect is crucial, as it underscores the potential for individuals to cultivate a positive relationship with their bodies by consciously engaging with and utilizing bodily sensations. More refined interoceptive processing—such as a greater attunement to internal bodily signals—can serve as a protective factor against negative mental health outcomes by improving emotional regulation [18], social functioning [19], and emotional connectivity [20]. The systematic review by [21] further highlights that greater interoceptive awareness is associated with better vagal tone, which in turn facilitates improved emotional regulation and psychological flexibility.

While a growing body of research investigates practices such as mindfulness training [22] or interoceptive technologies [23,24], this narrative review aims to consolidate this understanding by examining the specific influence of physical exercise on these diverse interoceptive dimensions. We propose that, as a particularly cost-effective and accessible intervention, the interplay between exercise and interoceptive processes offers a powerful pathway along the “inner road to happiness,” leading to a richer awareness of internal bodily signals and the cultivation of a range of adaptive behaviors, ultimately contributing to improved self-regulation and overall well-being. Indeed, this enhanced awareness can manifest in recognizing early signs of stress and responding proactively, making informed nutritional choices based on bodily cues, and utilizing exercise itself as a powerful tool for emotional regulation. This approach is supported by the existing literature, which is synthesized in this review.

### Methodology

This narrative review aims to synthesize the existing literature on the interplay between physical exercise and interoception, and its resultant impact on well-being. To this end, a comprehensive literature search was conducted primarily on PubMed using combinations of keywords such as “interoception,” “exercise,” “physical activity,” “well-being,” “mental health,” “emotional regulation,” and “self-regulation.” The selection of articles for inclusion was guided by their relevance to the review’s objectives, focusing on empirical studies, reviews, and influential theoretical papers. While no strict date limitations were applied to ensure the inclusion of foundational works, particular attention was given to recent advancements in the field. The selected literature was then synthesized through a thematic analysis, structured around three primary mechanisms through which exercise influences interoception: physiological changes, neurobiological adaptations, and psychological benefits. This approach allowed for a broad and insightful exploration, highlighting key findings and identifying directions for future research.

## 2. Interoceptive Responses to Exercise

Physical activity encompasses any bodily movement produced by skeletal muscles that results in energy expenditure; thus, we all engage in some level of physical activity for survival [25]. Walking or household tasks, for example, are forms of physical activity. However, the extent of physical activity we engage in is largely a matter of personal choice and can vary widely from person to person, as well as within an individual’s own routine over time, and during their lifetime. *Exercise* is a subset of physical activity, characterized by its planned, structured, and repetitive nature, and its goal of improving or maintaining physical fitness [25]. Exercise directly influences the afferent signals received by the interoceptive system through various mechanisms, including the induction of cardiovascular activation [26] and the initiation of a cascade of hormonal and metabolic responses [27,28]. Conversely, the regulation of physical exertion is under the constant oversight of the central nervous system, so that interoceptive mechanisms continuously monitor the physiological state of the body to maintain allostasis and guide goal-directed behavior [4,29].

Wallman-Jones and colleagues [30] highlight the intricate, bidirectional relationship between exercise and interoceptive processing. They emphasize that interoceptive feedback—particularly signals related to fatigue and effort—is key in regulating exercise intensity and duration, thus underscoring the dynamic interplay between physical activity and the body’s internal sensing.

**Figure 1:**
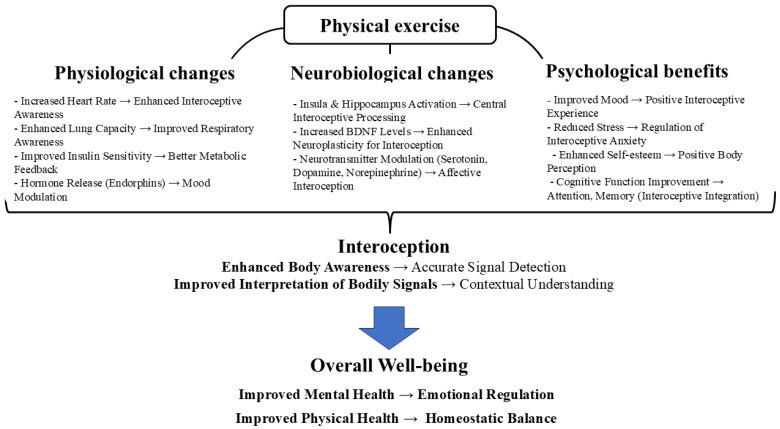
Integrated model of exercise influence on interoception and well-being.

To visually represent the multifaceted relationship between physical exercise and interoception, we propose a conceptual model in Figure 1 that illustrates the key pathways through which exercise impacts the various dimensions of interoceptive processes.

As depicted in Figure 1, physical exercise exerts influence through three primary mechanisms: physiological changes, neurobiological adaptations, and psychological benefits. These mechanisms converge to enhance interoception, which, in turn, contributes to improved overall well-being. Specifically, the figure details how physiological changes, such as increased heart rate and improved lung capacity, enhance the accuracy and awareness of inner-bodily signals; how neurobiological changes, including insula activation and BDNF increase, improve the central processing of interoceptive signals; and how psychological benefits, like reduced stress and enhanced mood, positively influence the interpretation of these signals. Ultimately, enhanced interoception fosters better emotional regulation and homeostatic balance, promoting overall mental and physical health.

A more detailed examination of these mechanisms will be presented in the subsequent sections.

## 3. The Physiological Effects of Exercise

Exercise elicits a wide range of physiological adaptations across multiple systems, including cardiovascular, respiratory, metabolic, muscular, hormonal, neurological, thermoregulatory, and stress response systems. These adaptations collectively contribute to improved health outcomes, enhanced physical fitness, and an elevated quality of life.

The most evident benefit of exercise is improved *cardiorespiratory fitness*, which can prevent cardiovascular disease [31]. Exercise, for example, improves the functionality of the endothelium (a layer of cells lining blood vessels), which plays a vital role in maintaining vascular homeostasis [32,33]. The endothelium regulates vascular tone, blood flow, and inflammatory responses. Exercise enhances its functionality by promoting the production of “nitric oxide”, a vasodilator that relaxes the smooth muscle cells in the vascular wall, leading to the widening of blood vessels (a process known as “vasodilation”). Improved vasodilation facilitates increased blood flow to various tissues, enhancing oxygen and nutrient delivery while promoting the removal of metabolic waste products. The ability of blood vessels to dilate in response to increased demand is crucial for maintaining cardiovascular health and function. The greatest benefit to this process is derived from regular moderate exercise, which promotes antioxidant status and preserves endothelial functionality [34]. A core component of these cardiovascular adaptations is the acute increase in heart rate during exercise, which has been shown to directly enhance interoceptive ability, suggesting that higher exertion levels lead to enhanced awareness of physiological signals [35]. This finding often stems from interventional studies where participants engage in acute exercise bouts while their interoceptive accuracy is measured [35]. This enhancement was observed across varying intensities of exercise, indicating a robust connection between heart rate increases and interoceptive sensibility.

*Respiratory efficiency* is also enhanced as regular exercise has been shown to significantly improve lung capacity [36]. Regular exercise habits can increase lung vital capacity, as the respiratory muscles become stronger and more efficient, allowing for greater lung expansion during inhalation. This increase in lung capacity is crucial for enhancing the overall efficiency of the respiratory system, as it allows for a greater volume of air to be inhaled and exhaled, facilitating better oxygen delivery to the bloodstream. While the direct link between exercise and respiratory interoception requires further investigation, observational studies have shown that awareness of respiratory signals is crucial for interoceptive accuracy. A study by [37], for example, found that individuals with heightened awareness of their breath exhibited differences in their ability to detect and discriminate respiratory resistive loads and maintain accurate perception of respiratory tidal volume, suggesting that training methods that enhance respiratory awareness, such as meditation, can improve interoceptive abilities. Therefore, it is plausible that the improved respiratory function from regular exercise, coupled with training focused on respiratory awareness, might lead to a greater sensibility and accuracy in perceiving internal respiratory changes.

The effects of exercise on *metabolic functions* are multifaceted, encompassing improvements in insulin sensitivity [38], alterations in adipose tissue metabolism [39], enhanced muscle substrate utilization, and favorable changes in energy balance. These adaptations collectively contribute to the prevention and management of metabolic disorders [40], highlighting the critical role of exercise in promoting overall health. Muscular adaptations include hypertrophy and increased strength, particularly through resistance training, which also enhances bone density, crucial for preventing osteoporosis [41]. Hormonal influences include the release of endorphins, improving mood and reducing pain perception. Exercise also modulates stress hormones like cortisol and enhances the balance of anabolic hormones, such as testosterone and growth hormone, vital for muscle repair and growth [42,43]. Critically, these metabolic changes are not merely physiological adaptations; they also significantly impact interoceptive processes, as the brain constantly monitors and interprets metabolic signals to maintain homeostasis and guide behavior [4,8,44].

Autonomic *thermoregulation* plays a key role in maintaining homeostasis during exercise, especially in response to the increased metabolic heat production. As core body temperature rises, physiological responses such as sweating and increased skin blood flow are triggered to dissipate heat and maintain thermal balance [45]. Following these responses, behavioral thermoregulation allows for conscious adjustments to further regulate body temperature. This involves seeking cooler environments or employing cooling strategies, such as drinking cold beverages. The heightened awareness of these autonomic and behavioral thermoregulatory processes during exercise, including the onset of sweating and changes in skin temperature, can significantly enhance interoceptive sensibility. This understanding often stems from both theoretical models and empirical observations in controlled laboratory settings. Enhanced interoceptive sensibility, particularly during heightened physiological states such as those experienced during physical exertion, can significantly refine one’s awareness of bodily sensations and improve self-regulation.

Thus, exercise elicits a wide range of physiological adaptations. These changes provide the brain with more precise and less noisy interoceptive signals. From an Active Inference perspective, this enhanced clarity in afferent information can reduce interoceptive prediction errors, allowing the brain to maintain more accurate internal models of the body’s state and better anticipate its needs. For instance, improved cardiovascular efficiency means the brain’s predictions about heart rate during exertion become more reliable, minimizing surprise and fostering a greater sense of bodily control.

## 4. Neurobiological Underpinnings of Exercise-Induced Interoception

At the *neurobiological level*, exercise provides a range of benefits that support brain health and cognitive function, including improved cerebral blood flow, reduced inflammation, and enhanced neuroplasticity [46]. Exercise stimulates the circulatory system, leading to a greater flow of blood to the brain and triggers the release of various neurochemicals, including neurotrophic factors that support the growth, survival, and differentiation of neurons, such as the Brain-Derived Neurotrophic Factor (BDNF). Critically, a rise in exercise-induced levels of BDNF has been found not only in structures primarily associated with motor functions, as the cerebellum, but also in other key brain areas, including the hippocampus [47], which is crucial for memory and cognitive functions [48]. The increase in BDNF levels in the hippocampus is particularly noteworthy, as it suggests that exercise may facilitate neuroplasticity and cognitive enhancement beyond mere motor coordination. This relationship underscores the potential of exercise to serve as a therapeutic strategy for cognitive decline and neurodegenerative conditions [49].

Various forms of exercise can influence interoceptive abilities, potentially through changes in brain regions involved in interoceptive processes, particularly the insula. For instance, an interventional study [50] reported enhanced functional connectivity in the bilateral anterior insula following an exercise intervention in older adults with Mild Cognitive Impairment. This increased connectivity was associated with improvements in subjective well-being, suggesting a potential neural mechanism through which exercise may promote psychological benefits. In addition, exercise training programs also induce increased connectivity within key brain networks, including the default mode network (DMN) and fronto-parietal cortex, which is coupled with enhanced executive functions and memory performance [51]. This enhanced connectivity throughout these networks facilitates a better integration of interoceptive information with emotional and cognitive processes, ultimately leading to improved self-awareness and emotional regulation [52].

Exercise has been demonstrated to have significant effects on the *neuroendocrine system*, particularly impacting the function of monoamine neurotransmitters. It can increase the availability of serotonin, a key regulator of mood and anxiety; dopamine, associated with motivation, reward, and pleasure; and norepinephrine, which plays a role in alertness, focus, and attention. These neurochemical changes are thought to underlie some of the positive effects of exercise on mood and cognitive function. For example, increased serotonin levels from exercise may enhance mood and reduce anxiety, facilitating a more accurate interpretation of bodily sensations [53,54]. Evidence for these neurochemical changes often comes from controlled experimental studies (e.g., involving acute exercise bouts) in both animal models and human participants.

Finally, exercise promotes *neuroplasticity*, the brain’s ability to adapt and reorganize itself [55], through various biological mechanisms, including the upregulation of neurotrophic factors such as the BDNF (as noted above) and insulin-like growth factor 1 (IGF-1), which play significant roles in neuronal survival, growth, and differentiation. Such plasticity enhances the brain’s capacity to process incoming signals, including the interoceptive ones, leading to improved emotional regulation and resilience to stress. Individuals with higher interoceptive awareness may exhibit greater adaptability in their emotional responses, which is highly beneficial for mental health.

From an Active Inference perspective, these neurobiological adaptations, such as enhanced connectivity and neuroplasticity, contribute to more precise internal models and optimized processing of interoceptive prediction errors, thereby improving the brain’s ability to accurately infer the body’s state and reduce uncertainty.

## 5. The Psychological Benefits of Exercise

In addition to its profound physiological and neurobiological effects, exercise also provides a wide range of psychological benefits that are key to promoting well-being.

### 5.1. Exercise and Stress Management

Regular physical activity is recognized as a significant enhancer of psychological resilience, empowering individuals to better manage stress and adversity [1,3,56]. This enhancement is supported by evidence demonstrating that exercise mitigates the physiological impact of stress. For instance, a review paper [2] highlighted the role of physical exercise in reducing COVID-19-related allostatic overload through neuroendocrine and immunological mechanisms, showcasing its benefits for both mental and physical well-being. Similarly, [57], an empirical study, found that higher aerobic fitness levels attenuate cardiovascular and salivary alpha amylase responses to acute psychosocial stress, further suggesting that regular aerobic exercise promotes physiological resilience to stress.

### 5.2. Exercise and Subjective Well-Being

The research study by Lin and colleagues [58] showed that the positive impact of exercise on subjective well-being is not direct, but rather mediated by two critical factors: the satisfaction of basic psychological needs and improved sleep quality. Specifically, exercise was found to foster a greater *sense of autonomy* (the feeling of control), *competence* (the perception of being effective), and *relatedness* (meaningful connections with others) among undergraduate students. These fulfilled needs, in turn, contribute significantly to increased subjective well-being. Furthermore, the study demonstrated that exercise promotes better sleep quality, which independently enhances feelings of well-being.

### 5.3. Exercise, Body Perception, and Self-Esteem

In their theoretical paper, Wallman-Jones et al. [59] present a compelling somatic-based model that elucidates the mechanisms through which physical activity can influence *self-esteem* via alterations in body perceptions. This framework is particularly significant as it moves beyond traditional psychological models, which often overlook the key role of bodily experiences in shaping self-concept. The authors propose that physical activity, by enhancing *body awareness* and fostering positive changes in *body perceptions*, directly contributes to increased self-esteem. Their model highlights the importance of interoceptive and proprioceptive feedback, suggesting that engaging in movement and experiencing physical competence can lead to a more positive evaluation of one’s body. Notably, this perspective offers a novel approach to understanding the psychological benefits of exercise, emphasizing the embodied nature of self-esteem.

### 5.4. Social Aspects of Exercise and Well-Being

Crucially, participating in physical activities often involves *social interaction*, which can enhance feelings of belonging and support, and further reinforce the embodied self-esteem discussed previously. This social aspect of exercise highlights how the act of moving and engaging with others can contribute to a stronger sense of self and connection. Group exercises or team sports provide opportunities for social engagement, fostering connections that contribute to improved mental health and emotional well-being. Structured physical activities have proven particularly beneficial for children with autism spectrum disorders, by increasing physical activity levels and also social interaction skills, providing a platform for practicing communication and social skills in a supportive environment [60].

### 5.5. Exercise and Cognitive Enhancement: Supporting Interoception and Well-Being

Enhanced *cognitive capabilities*, facilitated by physical activity, can indirectly influence interoceptive awareness and processing, serving a supportive role in overall psychological well-being. A key aspect of enhanced cognitive function is improved attentional control, which allows individuals to more effectively focus on and interpret internal bodily signals. Wallman-Jones et al. [35] investigated whether increases in interoceptive ability, which may occur following acute physical activity conditions, could be further enhanced by directing attention towards interoceptive signals during the activity. The study, employing incremental cycling intensities, found that engaging in acute physical activity led to an increase in interoceptive accuracy. However, the anticipated enhancement in interoceptive accuracy through directed attention was not observed, suggesting that simply directing focus may not offer additional benefits to exercise’s influence on interoception.

Better executive functions, such as *cognitive flexibility* and *inhibitory control*, contribute to the ability to differentiate between relevant and irrelevant interoceptive cues, reducing noise and enhancing signal detection. For example, individuals with improved cognitive flexibility might be better able to adapt their interoceptive interpretations in response to changing contexts, such as distinguishing between exercise-induced heart rate elevation and anxiety-related tachycardia [61,62].

The relationship between regular physical activity and cognitive performance has garnered considerable attention within the field of sports science. Numerous studies support the premise that engaging in structured exercise not only enhances physical capabilities but also contributes to cognitive enhancements, particularly in areas such as *executive functioning*, *memory*, and overall *cognitive resilience*. Athletes, especially those in disciplines demanding high levels of skill and concentration, often demonstrate advantageous cognitive capabilities compared to non-athletes or novices in their respective sports. For example, expert climbers—who must navigate complex environments, assess distances, and plan movements—exhibit enhanced visuospatial memory compared to novices [63]. This is typically observed in comparative observational studies. Critically, these benefits are not limited to athletic populations; appreciable effects are also observed in non-athletes, highlighting the universal relevance of exercise for cognitive processes. For example, [64], a quasi-experimental study, investigated the benefits of exercise on cognitive and motor functions in individuals with cardiovascular risk factors utilizing a dual-task walking paradigm and fNIRS. Their findings suggest that exercise may play an essential role in maintaining cognitive and motor functions and potentially contribute to the prevention of cognitive decline. A comprehensive meta-analysis assessed the impact of exercise interventions on cognitive function in older adults, revealing significant positive effects on memory, attention, and executive functions [65].

In the context of children [66], a rigorous six-month randomized controlled trial in overweight children revealed significant enhancements in executive functions (working memory, cognitive flexibility, and inhibitory control) following sustained aerobic exercise (see also [67]).

Overall, this body of research indicates that regular exercise serves as a crucial component not only for physical health but also for mental and cognitive well-being, fostering improved decision-making abilities across various populations. From an Active Inference perspective, these psychological benefits—such as reduced stress, improved mood, and enhanced self-esteem—contribute to a reduction in the “noise” or uncertainty of interoceptive signals. This, in turn, allows for a more accurate and less threatening interpretation of internal bodily states, minimizing prediction errors related to internal distress and fostering a more coherent and adaptive internal model of the self.

## 6. General Discussion

Our analysis highlights that physical exercise is a powerful and multifaceted tool for enhancing interoception and, consequently, promoting well-being. Through its physiological, neurobiological, and psychological effects, exercise emerges as a crucial intervention to improve interoceptive functions and foster adaptive behaviors. This relationship can be understood through the lens of Active Inference [8,9,68,69], which proposes that the brain actively predicts and updates its internal states. In this perspective, we suggest that exercise can influence this process in several key ways. Improved physiological signaling may provide more precise data for the brain’s internal models, neurobiological adaptations may enhance the computational capacity for prediction and error processing (e.g., through insula connectivity and BDNF-mediated plasticity), and psychological benefits may reduce the “noise” of distress, allowing for a more accurate interpretation of interoceptive cues [70,71]. Together, these mechanisms may contribute to a more optimized and adaptive interoceptive inference, reducing uncertainty about internal bodily states and fostering a more coherent sense of self and well-being.

### 6.1. Broader Implications and Future Directions

The therapeutic potential of exercise training to mitigate aging’s effects and promote health is gaining significant attention [46]. Exercise’s ability to modulate endocrine function towards a more youthful state and attenuate biological aging biomarkers further underscores its importance. Given this, cultivating an active lifestyle from childhood to old age represents a potent strategy for fostering lifelong well-being, promoting a positive body image, and establishing a foundation for sustained health.

A crucial area for future inquiry lies in understanding how to optimize exercise benefits for different populations [72]. Investigating the synergistic effects of combined cognitive and physical training, or the integration of mindfulness into physical activity, could offer valuable insights for interoceptive enhancement [73]. Crucially, understanding and delineating optimal types and amounts of exercise tailored to individual needs remains essential. Developing personalized exercise prescriptions that consider age, health status, and personal preferences will be pivotal in maximizing the benefits of physical activity on interoceptive processes and overall well-being [74].

Recognizing these profound cognitive and interoceptive benefits, a critical question becomes how to most effectively facilitate the widespread adoption of exercise across diverse populations. Modifying urban environments to make exercise more accessible and enjoyable presents a compelling strategy. For instance, specific urban design interventions, such as creating green spaces and pedestrian-friendly areas, can significantly increase exercise levels among urban residents, leading to measurable improvements in physical and mental health outcomes [75,76]. This highlights the crucial interplay between environmental design and the promotion of a more active lifestyle.

### 6.2. Exercise as a Cost-Effective and Synergistic Therapeutic Strategy

Despite accumulating evidence supporting the multifaceted benefits of exercise, it remains underutilized as a cost-effective intervention for enhancing mental and physical health. This is particularly concerning given its potential as an adjunctive or alternative treatment for various conditions, such as addiction recovery [77].

The role of exercise as a potent interoceptive intervention becomes even clearer when compared with other practices like mindfulness and biofeedback. Recent research provides valuable insights, suggesting that all three approaches can be similarly effective in promoting well-being [78,79,80]. While these modalities engage distinct mechanisms, a growing body of evidence suggests a synergistic relationship between mindfulness and exercise [81]. Mindfulness, for example, can positively impact physical health by promoting healthier lifestyle choices and facilitating more consistent engagement in physical activities [82]. Contextual factors significantly influence the efficacy of these interventions; for individuals with sedentary lifestyles, mindfulness strategies can be a guide to increase overall enjoyment of exercise, thereby improving adherence [83,84]. A study even showed that participants who engaged in mindfulness-based physical activity experienced greater overall satisfaction than those who undertook traditional programs [85]. In conclusion, while both exercise and mindfulness possess intrinsic value, integrating the two holds great promise for enhancing overall effectiveness.

### 6.3. Limitations and Open Questions

While our analysis highlights the significant potential of physical exercise as an interoceptive intervention, it is essential to acknowledge the limitations inherent in the current body of research. A primary constraint is that much of the cited evidence, particularly concerning the intricate neurobiological pathways linking exercise to interoception, remains largely correlational or stems from early-phase studies, thus posing challenges for inferring direct causality [86].

Furthermore, the relationship between exercise and interoception—particularly regarding the effects on interoceptive accuracy—has been a subject of investigation with complex and sometimes inconsistent findings. While some studies have explored whether regular exercise leads to improvements in cardiac interoceptive processing, the evidence remains mixed. For instance, Yoris et al. [87] and Amaya et al. [88] found no definitive evidence that habitual physical activity significantly enhances cardiac interoceptive accuracy or provides conclusive benefits to cognitive functions during exercise. The inconsistencies in these outcomes can stem from varying methodologies, types of interoceptive tasks used, and participant characteristics [89,90]. This suggests that while there is theoretical support for the benefits of exercise on interoception, the practical evidence depicts a more nuanced landscape, where effects may not be as universally applicable as previously assumed [52,87]. Another important limitation is the broad conceptualization of interoception in this review, which did not delve into the specific and differential effects of exercise on its various dimensions (e.g., interoceptive accuracy, sensibility, or awareness). Additionally, many widely used interoception measures, such as heartbeat detection tasks, can sometimes lack ecological validity, failing to fully capture the dynamic, multimodal, and context-dependent nature of interoceptive experiences in real-world scenarios [91,92].

Therefore, future research must strive to clarify these mixed results by standardizing methodologies, broadening participant demographics, and utilizing more rigorous experimental designs and ecologically valid measures. Such efforts will be essential for translating research findings into more effective, evidence-based interventions and for truly understanding the dynamic interplay between exercise and interoception.

## 7. Conclusions

This narrative review highlights the pivotal role of physical exercise as a catalyst for enhancing interoceptive functions and promoting overall well-being. By influencing physiological, neurobiological, and psychological processes, exercise effectively fosters a richer understanding of internal bodily states and supports adaptive behaviors. To unlock its full therapeutic potential, it is essential to adopt personalized and integrated strategies.

### Recommendations

Based on the evidence analyzed, targeted recommendations emerge for advancing both research and practice in leveraging exercise for interoceptive enhancement and general well-being. Future research must prioritize the development and validation of personalized exercise interventions, not only for populations with interoceptive dysfunctions, but also to improve the well-being of the general population. This requires systematically investigating optimal modalities, intensities, and durations, as well as the precise neurobiological mechanisms involved. At a practical and professional level, figures such as coaches, personal trainers, and therapists should integrate personalized exercise prescriptions, informed by an individual’s interoceptive profile, to improve the effectiveness of their interventions. This can include incorporating mindfulness-based movement practices or biofeedback techniques. On a broader public health and policy level, it is crucial to promote exercise not only for physical fitness, but also for its profound cognitive and interoceptive benefits. This entails advocating for funding in urban planning, launching public health campaigns that emphasize the mental well-being aspects of exercise, and implementing school-based programs that combine physical education with elements of interoceptive awareness and self-regulation.

## Data Availability

The original contributions presented in this study are included in the article. Further inquiries can be directed to the corresponding author.

## References

[1] Barca L., Iodice P., Chaigneau A., Lancia G., Pezzulo G. (2025). Emotional Distress and Affective Knowledge Representation One Year after the COVID-19 Outbreak. PLoS ONE.

[2] Eöry A., Békési D., Eöry A., Rózsa S. (2021). Physical Exercise as a Resilience Factor to Mitigate COVID-Related Allostatic Overload. Psychother. Psychosom..

[3] Maugeri G., Castrogiovanni P., Battaglia G., Pippi R., D’Agata V., Palma A., Di Rosa M., Musumeci G. (2020). The Impact of Physical Activity on Psychological Health during COVID-19 Pandemic in Italy. Heliyon.

[4] Tschantz A., Barca L., Maisto D., Buckley C.L., Seth A.K., Pezzulo G. (2022). Simulating Homeostatic, Allostatic and Goal-Directed Forms of Interoceptive Control Using Active Inference. Biol. Psychol..

[5] Feldman M.J., Bliss-Moreau E., Lindquist K.A. (2024). The Neurobiology of Interoception and Affect. Trends Cogn. Sci..

[6] Garfinkel S.N., Seth A.K., Barrett A.B., Suzuki K., Critchley H.D. (2015). Knowing Your Own Heart: Distinguishing Interoceptive Accuracy from Interoceptive Awareness. Biol. Psychol..

[7] Garfinkel S.N., Manassei M.F., Hamilton-Fletcher G., In den Bosch Y., Critchley H.D., Engels M. (2016). Interoceptive Dimensions across Cardiac and Respiratory Axes. Philos. Trans. R. Soc. B Biol. Sci..

[8] Pezzulo G., Rigoli F., Friston K. (2015). Active Inference, Homeostatic Regulation and Adaptive Behavioural Control. Prog. Neurobiol..

[9] Parr T., Pezzulo G., Friston K.J. (2022). Active Inference: The Free Energy Principle in Mind, Brain, and Behavior.

[10] Barrett L.F., Bar M. (2009). See It with Feeling: Affective Predictions during Object Perception. Philos. Trans. R. Soc. B Biol. Sci..

[11] Gu X., Hof P.R., Friston K.J., Fan J. (2013). Anterior Insular Cortex and Emotional Awareness. J. Comp. Neurol..

[12] Biddell H., Solms M., Slagter H., Laukkonen R. (2024). Arousal Coherence, Uncertainty, and Well-Being: An Active Inference Account. Neurosci. Conscious..

[13] Smith R., Varshney L.R., Nagayama S., Kazama M., Kitagawa T., Ishikawa Y. (2022). A Computational Neuroscience Perspective on Subjective Wellbeing within the Active Inference Framework. Int. J. Wellbeing.

[14] Kiverstein J., Miller M. (2023). Playfulness and the Meaningful Life: An Active Inference Perspective. Neurosci. Conscious..

[15] Clemente R., Murphy A., Murphy J. (2024). The Relationship between Self-Reported Interoception and Anxiety: A Systematic Review and Meta-Analysis. Neurosci. Biobehav. Rev..

[16] Barca L., Pezzulo G. (2020). Keep Your Interoceptive Streams under Control: An Active Inference Perspective on Anorexia Nervosa. Cogn. Affect. Behav. Neurosci..

[17] Barca L., Maisto D., Pezzulo G. (2023). Modeling and Controlling the Body in Maladaptive Ways: An Active Inference Perspective on Non-Suicidal Self-Injury Behaviors. Neurosci. Conscious..

[18] Montoya-Hurtado O.L., Sobral-Monteiro-Junior R., Meneses-Castaño C.Y., Sancho-Sánchez C., Martínez-Sabater A., Andrés-Olivera P., Sanchez-Conde P., Sánchez-Toledo J.P., Criado-Gutiérrez J.M., Criado-Pérez L. (2024). Body Awareness as a Protective Factor against Suicidal Orientations in College Students. Behav. Sci..

[19] Feldman M.J., MacCormack J.K., Bonar A.S., Lindquist K.A. (2023). Interoceptive Ability Moderates the Effect of Physiological Reactivity on Social Judgment. Emotion.

[20] Tajadura-Jiménez A., Tsakiris M. (2014). Balancing the “Inner” and the “Outer” Self: Interoceptive Sensitivity Modulates Self–Other Boundaries. J. Exp. Psychol. Gen..

[21] Edwards D.J., Pinna T. (2020). A Systematic Review of Associations Between Interoception, Vagal Tone, and Emotional Regulation: Potential Applications for Mental Health, Wellbeing, Psychological Flexibility, and Chronic Conditions. Front. Psychol..

[22] Weng H.Y., Feldman J.L., Leggio L., Napadow V., Park J., Price C.J. (2021). Interventions and Manipulations of Interoception. Trends Neurosci..

[23] Schoeller F., Horowitz A.H., Jain A., Maes P., Reggente N., Christov-Moore L., Pezzulo G., Barca L., Allen M., Salomon R. (2024). Interoceptive Technologies for Psychiatric Interventions: From Diagnosis to Clinical Applications. Neurosci. Biobehav. Rev..

[24] Schoeller F., Haar A.J.H., Jain A., Maes P. (2019). Enhancing Human Emotions with Interoceptive Technologies. Phys. Life Rev..

[25] Caspersen C.J., Powell K.E., Christenson G.M. (1985). Physical Activity, Exercise, and Physical Fitness: Definitions and Distinctions for Health-Related Research. Public Health Rep..

[26] Wilson J.R. (1987). Exercise and the Failing Heart. Cardiol. Clin..

[27] Sellami M., Bragazzi N.L., Slimani M., Hayes L., Jabbour G., De Giorgio A., Dugué B. (2019). The Effect of Exercise on Glucoregulatory Hormones: A Countermeasure to Human Aging: Insights from a Comprehensive Review of the Literature. Int. J. Environ. Res. Public Health.

[28] Winder W.W., Hickson R.C., Hagberg J.M., Ehsani A.A., McLane J.A. (1979). Training-Induced Changes in Hormonal and Metabolic Responses to Submaximal Exercise. J. Appl. Physiol..

[29] Craig A.D. (2003). Interoception: The Sense of the Physiological Condition of the Body. Curr. Opin. Neurobiol..

[30] Wallman-Jones A., Perakakis P., Tsakiris M., Schmidt M. (2021). Physical Activity and Interoceptive Processing: Theoretical Considerations for Future Research. Int. J. Psychophysiol..

[31] Isath A., Koziol K.J., Martinez M.W., Garber C.E., Martinez M.N., Emery M.S., Baggish A.L., Naidu S.S., Lavie C.J., Arena R. (2023). Exercise and Cardiovascular Health: A State-of-the-Art Review. Prog. Cardiovasc. Dis..

[32] Joyner M.J., Green D.J. (2009). Exercise Protects the Cardiovascular System: Effects beyond Traditional Risk Factors. J. Physiol..

[33] Pedralli M.L., Marschner R.A., Kollet D.P., Neto S.G., Eibel B., Tanaka H., Lehnen A.M. (2020). Different Exercise Training Modalities Produce Similar Endothelial Function Improvements in Individuals with Prehypertension or Hypertension: A Randomized Clinical Trial Exercise, Endothelium and Blood Pressure. Sci. Rep..

[34] Di Francescomarino S., Sciartilli A., Di Valerio V., Di Baldassarre A., Gallina S. (2009). The Effect of Physical Exercise on Endothelial Function. Sports Med..

[35] Wallman-Jones A., Palser E.R., Benzing V., Schmidt M. (2022). Acute Physical-Activity Related Increases in Interoceptive Ability Are Not Enhanced with Simultaneous Interoceptive Attention. Sci. Rep..

[36] Li L.K., Cassim R., Perret J.L., Dharmage S.C., Lowe A.J., Lodge C.J., Russell M.A. (2023). The Longitudinal Association between Physical Activity, Strength and Fitness, and Lung Function: A UK Biobank Cohort Study. Respir. Med..

[37] Daubenmier J., Sze J., Kerr C.E., Kemeny M.E., Mehling W. (2013). Follow Your Breath: Respiratory Interoceptive Accuracy in Experienced Meditators. Psychophysiology.

[38] Hawley J.A., Lessard S.J. (2008). Exercise Training-Induced Improvements in Insulin Action. Acta Physiol..

[39] Muscella A., Stefàno E., Lunetti P., Capobianco L., Marsigliante S. (2020). The Regulation of Fat Metabolism During Aerobic Exercise. Biomolecules.

[40] Sampath Kumar A., Maiya A.G., Shastry B.A., Vaishali K., Ravishankar N., Hazari A., Gundmi S., Jadhav R. (2019). Exercise and Insulin Resistance in Type 2 Diabetes Mellitus: A Systematic Review and Meta-Analysis. Ann. Phys. Rehabil. Med..

[41] Green D.J., Chasland L.C., Yeap B.B., Naylor L.H. (2024). Comparing the Impacts of Testosterone and Exercise on Lean Body Mass, Strength and Aerobic Fitness in Aging Men. Sports Med.—Open.

[42] Childs E., de Wit H. (2014). Regular Exercise Is Associated with Emotional Resilience to Acute Stress in Healthy Adults. Front. Physiol..

[43] Hare B.D., Beierle J.A., Toufexis D.J., Hammack S.E., Falls W.A. (2014). Exercise-Associated Changes in the Corticosterone Response to Acute Restraint Stress: Evidence for Increased Adrenal Sensitivity and Reduced Corticosterone Response Duration. Neuropsychopharmacology.

[44] Fotopoulou A., Tsakiris M. (2017). Mentalizing Homeostasis: The Social Origins of Interoceptive Inference. Neuropsychoanalysis.

[45] Périard J.D., Eijsvogels T.M.H., Daanen H.A.M. (2021). Exercise under Heat Stress: Thermoregulation, Hydration, Performance Implications, and Mitigation Strategies. Physiol. Rev..

[46] Tari A.R., Walker T.L., Huuha A.M., Sando S.B., Wisloff U. (2025). Neuroprotective Mechanisms of Exercise and the Importance of Fitness for Healthy Brain Ageing. Lancet.

[47] Cotman C.W., Berchtold N.C. (2002). Exercise: A Behavioral Intervention to Enhance Brain Health and Plasticity. Trends Neurosci..

[48] Gomez-Pinilla F., Zhuang Y., Feng J., Ying Z., Fan G. (2011). Exercise Impacts Brain-Derived Neurotrophic Factor Plasticity by Engaging Mechanisms of Epigenetic Regulation. Eur. J. Neurosci..

[49] Augusto-Oliveira M., Arrifano G.P., Leal-Nazaré C.G., Santos-Sacramento L., Lopes-Araújo A., Royes L.F.F., Crespo-Lopez M.E. (2023). Exercise Reshapes the Brain: Molecular, Cellular, and Structural Changes Associated with Cognitive Improvements. Mol. Neurobiol..

[50] Won J., Nielson K.A., Smith J.C. (2022). Subjective Well-Being and Bilateral Anterior Insula Functional Connectivity After Exercise Intervention in Older Adults With Mild Cognitive Impairment. Front. Neurosci..

[51] Won J., Nielson K.A., Smith J.C. (2023). Large-Scale Network Connectivity and Cognitive Function Changes After Exercise Training in Older Adults with Intact Cognition and Mild Cognitive Impairment. J. Alzheimer’s Dis. Rep..

[52] Paulus M.P., Stewart J.L., Haase L. (2013). Treatment Approaches for Interoceptive Dysfunctions in Drug Addiction. Front. Psychiatry.

[53] Kandola A., Ashdown-Franks G., Hendrikse J., Sabiston C.M., Stubbs B. (2019). Physical Activity and Depression: Towards Understanding the Antidepressant Mechanisms of Physical Activity. Neurosci. Biobehav. Rev..

[54] Ross R.E., VanDerwerker C.J., Saladin M.E., Gregory C.M. (2023). The Role of Exercise in the Treatment of Depression: Biological Underpinnings and Clinical Outcomes. Mol. Psychiatry.

[55] Hötting K., Röder B. (2013). Beneficial Effects of Physical Exercise on Neuroplasticity and Cognition. Neurosci. Biobehav. Rev..

[56] Xin S., Ma X. (2023). Mechanisms of Physical Exercise Effects on Anxiety in Older Adults during the COVID-19 Lockdown: An Analysis of the Mediating Role of Psychological Resilience and the Moderating Role of Media Exposure. Int. J. Environ. Res. Public Health.

[57] Wyss T., Boesch M., Roos L., Tschopp C., Frei K.M., Annen H., La Marca R. (2016). Aerobic Fitness Level Affects Cardiovascular and Salivary Alpha Amylase Responses to Acute Psychosocial Stress. Sports Med.—Open.

[58] Lin S., Li L., Zheng D., Jiang L. (2022). Physical Exercise and Undergraduate Students’ Subjective Well-Being: Mediating Roles of Basic Psychological Need Satisfaction and Sleep Quality. Behav. Sci..

[59] Wallman-Jones A., Eigensatz M., Rubeli B., Schmidt M., Benzing V. (2024). The Importance of Body Perception in the Relationship between Physical Activity and Self-Esteem in Adolescents. Int. J. Sport Exerc. Psychol..

[60] Xing Y., Huang S., Zhao Y., Wu X. (2025). Effects of Group Sports Activities on Physical Activity and Social Interaction Abilities of Children with Autism Spectrum Disorders. Front. Psychol..

[61] Maisto D., Barca L., Van den Bergh O., Pezzulo G. (2021). Perception and Misperception of Bodily Symptoms from an Active Inference Perspective: Modelling the Case of Panic Disorder. Psychol. Rev..

[62] Pezzulo G., Maisto D., Barca L., Bergh O.V. (2019). den Symptom Perception from a Predictive Processing Perspective. Clin. Psychol. Eur..

[63] Pezzulo G., Barca L., Bocconi A.L., Borghi A.M. (2010). When Affordances Climb into Your Mind: Advantages of Motor Simulation in a Memory Task Performed by Novice and Expert Rock Climbers. Brain Cogn..

[64] Talamonti D., Vincent T., Fraser S., Nigam A., Lesage F., Bherer L. (2021). The Benefits of Physical Activity in Individuals with Cardiovascular Risk Factors: A Longitudinal Investigation Using fNIRS and Dual-Task Walking. J. Clin. Med..

[65] Araque-Martínez M.Á., Artés-Rodríguez E.M., Ruiz-Montero P.J., Casimiro-Andújar A.J. (2021). Physical, Cognitive and Emotional Outcomes in Older Adults Exercisers: A Systematic Review. J. Hum. Sport Exerc..

[66] Davis C.L., Tomporowski P.D., McDowell J.E., Austin B., Miller P.H., Yanasak N.E., Allison J.D., Naglieri J.A. (2011). Exercise Improves Executive Function and Achievement and Alters Brain Activation in Overweight Children: A Randomized, Controlled Trial. Health Psychol..

[67] Shao X., Tan L.H., He L. (2022). Physical Activity and Exercise Alter Cognitive Abilities, and Brain Structure and Activity in Obese Children. Front. Neurosci..

[68] Pezzulo G., Barca L., Friston K.J. (2015). Active Inference and Cognitive-Emotional Interactions in the Brain. Behav. Brain Sci..

[69] Seth A.K. (2013). Interoceptive Inference, Emotion, and the Embodied Self. Trends Cogn. Sci. (Regul. Ed.).

[70] Barrett L.F. (2017). The Theory of Constructed Emotion: An Active Inference Account of Interoception and Categorization. Soc. Cogn. Affect. Neurosci..

[71] Paulus M.P., Feinstein J.S., Khalsa S.S. (2019). An Active Inference Approach to Interoceptive Psychopathology. Annu. Rev. Clin. Psychol..

[72] Dawe J., Cavicchiolo E., Palombi T., Baiocco R., Antoniucci C., Pistella J., Alessandri G., Filosa L., Tavolucci S., Borghi A.M. (2024). Measuring Self-Efficacy for Exercise among Older Adults: Psychometric Properties and Measurement Invariance of a Brief Version of the Self-Efficacy for Exercise (SEE) Scale. Healthcare.

[73] Anguera J.A., Volponi J.J., Simon A.J., Gallen C.L., Rolle C.E., Anguera-Singla R., Pitsch E.A., Thompson C.J., Gazzaley A. (2022). Integrated Cognitive and Physical Fitness Training Enhances Attention Abilities in Older Adults. npj Aging.

[74] Shimura A., Masuya J., Yokoi K., Morishita C., Kikkawa M., Nakajima K., Chen C., Nakagawa S., Inoue T. (2023). Too Much Is Too Little: Estimating the Optimal Physical Activity Level for a Healthy Mental State. Front. Psychol..

[75] Ruotolo F., Rapuano M., Masullo M., Maffei L., Ruggiero G., Iachini T. (2024). Well-Being and Multisensory Urban Parks at Different Ages: The Role of Interoception and Audiovisual Perception. J. Environ. Psychol..

[76] Bogdanov V.B., Marquis-Favre C., Cottet M., Beffara B., Perrin F., Dumortier D., Ellermeier W. (2022). Nature and the City: Audiovisual Interactions in Pleasantness and Psychophysiological Reactions. Appl. Acoust..

[77] Lynch W.J., Peterson A.B., Sanchez V., Abel J., Smith M.A. (2013). Exercise as a Novel Treatment for Drug Addiction: A Neurobiological and Stage-Dependent Hypothesis. Neurosci. Biobehav. Rev..

[78] Thompson T.P., Horrell J., Taylor A.H., Wanner A., Husk K., Wei Y., Creanor S., Kandiyali R., Neale J., Sinclair J. (2020). Physical Activity and the Prevention, Reduction, and Treatment of Alcohol and Other Drug Use across the Lifespan (The PHASE Review): A Systematic Review. Ment. Health Phys. Act..

[79] van der Zwan J.E., de Vente W., Huizink A.C., Bögels S.M., de Bruin E.I. (2015). Physical Activity, Mindfulness Meditation, or Heart Rate Variability Biofeedback for Stress Reduction: A Randomized Controlled Trial. Appl. Psychophysiol. Biofeedback.

[80] de Bruin E.I., van der Zwan J.E., Bögels S.M. (2016). A RCT Comparing Daily Mindfulness Meditations, Biofeedback Exercises, and Daily Physical Exercise on Attention Control, Executive Functioning, Mindful Awareness, Self-Compassion, and Worrying in Stressed Young Adults. Mindfulness.

[81] Yıldız M.E., Günel İ., Dalbudak İ. (2024). The Relationship between Physical Activity and Mindful Awareness of University Students. Phys. Educ. Stud..

[82] Loucks E.B., Schuman-Olivier Z., Britton W.B., Fresco D.M., Desbordes G., Brewer J.A., Fulwiler C. (2015). Mindfulness and Cardiovascular Disease Risk: State of the Evidence, Plausible Mechanisms, and Theoretical Framework. Curr. Cardiol. Rep..

[83] Ruffault A., Bernier M., Juge N., Fournier J.F. (2016). Mindfulness May Moderate the Relationship Between Intrinsic Motivation and Physical Activity: A Cross-Sectional Study. Mindfulness.

[84] Liguori F., Calella P. (2024). Physical Activity and Wellbeing in Prisoners: A Scoping Review. Int. J. Prison. Health.

[85] Sala M., Geary B., Baldwin A.S. (2021). A Mindfulness-Based Physical Activity Intervention: A Randomized Pilot Study. Psychosom. Med..

[86] Poldrack R.A. (2006). Can Cognitive Processes Be Inferred from Neuroimaging Data?. Trends Cogn. Sci..

[87] Yoris A.E., Cira L.F., Luque-Casado A., Salvotti C., Tajadura-Jiménez A., Avancini C., Zarza-Rebollo J.A., Sanabria D., Perakakis P. (2024). Delving into the Relationship between Regular Physical Exercise and Cardiac Interoception in Two Cross-Sectional Studies. Neuropsychologia.

[88] Amaya Y., Abe T., Kanbara K., Shizuma H., Akiyama Y., Fukunaga M. (2021). The Effect of Aerobic Exercise on Interoception and Cognitive Function in Healthy University Students: A Non-Randomized Controlled Trial. BMC Sports Sci. Med. Rehabil..

[89] Brevers D., Billieux J., de Timary P., Desmedt O., Maurage P., Perales J.C., Suárez-Suárez S., Bechara A. (2024). Physical Exercise to Redynamize Interoception in Substance Use Disorders. Curr. Neuropharmacol..

[90] Mulder J., Boelens M., van der Velde L.A., Brust M., Kiefte-de Jong J.C. (2025). The Role of Interoception in Lifestyle Factors: A Systematic Review. Neurosci. Biobehav. Rev..

[91] Garfinkel S.N., Schulz A., Tsakiris M. (2022). Addressing the Need for New Interoceptive Methods. Biol. Psychol..

[92] Brener J., Ring C. (2016). Towards a Psychophysics of Interoceptive Processes: The Measurement of Heartbeat Detection. Philos. Trans. R. Soc. B Biol. Sci..

